# Establishing Differences in Thermographic Patterns between the Various Complications in Diabetic Foot Disease

**DOI:** 10.1155/2018/9808295

**Published:** 2018-03-12

**Authors:** Alfred Gatt, Owen Falzon, Kevin Cassar, Christian Ellul, Kenneth P. Camilleri, Jean Gauci, Stephen Mizzi, Anabelle Mizzi, Cassandra Sturgeon, Liberato Camilleri, Nachiappan Chockalingam, Cynthia Formosa

**Affiliations:** ^1^Faculty of Health Sciences, University of Malta, Msida, Malta; ^2^Centre for Biomedical Cybernetics, Faculty of Engineering, University of Malta, Msida, Malta; ^3^Faculty of Medicine and Surgery, University of Malta, Msida, Malta; ^4^Department of Systems & Control Engineering, University of Malta, Msida, Malta; ^5^Department of Statistics and Operations Research, Faculty of Science, University of Malta, Msida, Malta; ^6^Faculty of Health Science, Staffordshire University, Stoke-on-Trent, UK

## Abstract

**Aim:**

To evaluate the potential of thermography as an assessment tool for the detection of foot complications by understanding the variations in temperature that occur in type 2 diabetes mellitus (DM).

**Methods:**

Participants were categorized according to a medical examination, ankle brachial index, doppler waveform analysis, and 10-gram monofilament testing into five groups: healthy adult, DM with no complications, DM with peripheral neuropathy, DM with neuroischaemia, and DM with peripheral arterial disease (PAD) groups. Thermographic imaging of the toes and forefeet was performed.

**Results:**

43 neuroischaemic feet, 41 neuropathic feet, 58 PAD feet, 21 DM feet without complications, and 126 healthy feet were analyzed. The temperatures of the feet and toes were significantly higher in the complications group when compared to the healthy adult and DM healthy groups. The higher the temperatures of the foot in DM, the higher the probability that it is affected by neuropathy, neuroischaemia, or PAD.

**Conclusions:**

Significant differences in mean temperatures exist between participants who were healthy and those with DM with no known complications when compared to participants with neuroischaemia, neuropathy, or PAD. As foot temperature rises, so does the probability of the presence of complications of neuropathy, neuroischaemia, or peripheral arterial disease.

## 1. Introduction

The diabetic foot is characterized by the presence of various complications that typically tend to develop as a result of poor glycaemic control. The main complications include neuropathy, peripheral arterial disease (PAD), and neuroischaemia, amongst others. These complications are amongst the most serious and costly as they often lead to amputation. In many cases, development of diabetic foot complications can be avoided or substantially delayed with timely assessment, diagnosis, and treatment provided at an early stage of the disease. Prophylactic foot care has been shown to decrease patient morbidity [1].

Common diabetic foot complications such as ischaemia and neuropathy have an effect on the temperature of the foot [2]. It is postulated that changes in temperature of the foot may be indicative of the presence of such complications.

Thermography or medical infrared imaging may be used to detect temperature changes. This technique is deemed safe since it is noncontact, noninvasive, and nonirradiant and has been utilized in a number of medical applications including imaging of the breast [3], skin [4] and foot vascular complications, and ulceration in diabetes [5–7].

The current clinical practice of temperature assessment is mainly by manual palpation of foot temperature. However, a gradual increase in foot temperature may be too subtle to be detected only by the hand, making the timely and early detection of underlying pathology difficult. It has been established that increased temperatures in the foot may be present up to a week before a foot ulcer occurs [8], thus making it important for any variations in temperature to be detected promptly.

Thermal imaging offers an excellent means of making a quantitative determination of surface temperature and can offer an alternative mode of detection of major foot complications since it has been reported that monitoring of skin temperature reduces the risk of diabetic foot ulceration in high-risk patients [9].

Although Nagase et al. had looked at variations in plantar thermographic patterns in normal controls and nonulcer diabetic patients, it has not been fully elucidated to what extent the individual variations of the plantar thermographic patterns show different trends between these two cohorts [10].

Presently, little is known about the range of abnormal thermoregulation in those patients with diabetes presenting both for screening and management [8]. The aim of this study was to evaluate the potential of thermography as an assessment tool for the detection of type 2 diabetic foot disease by assessing the variations in temperature that occur in the diabetic foot before ulceration appears. Objectives were to find a possible correlation between temperature readings of the plantar foot and toes in diabetic foot complications to reduce the risk of foot ulceration by early detection of pathologies.

## 2. Method

This study employed medical infrared imaging to visualize the temperature distribution of the feet of participants living with DM with or without complications and healthy controls. Following ethical approval from the University Ethics Committee, initially, healthy adult participants were recruited, medically examined, and imaged as reported elsewhere [11] whilst participants with type 2 diabetes mellitus were recruited from the patient list of a vascular surgeon. All participants underwent a thorough clinical examination that included validated tests for neuropathy [12] and peripheral arterial disease [13].

Participants with DM were categorized into 4 groups based on the medical examination and testing: a heathy DM group (i.e., presenting with DM but no significant medical comorbidities and/or complications), a PAD group (presenting with ABPI < 0.6 and monophasic Doppler spectral waveforms at the ankles, but no neuropathy), a neuropathic group (presenting with positive 10-gram monofilament at any one of 10 tested sites and/or reduced vibration perception threshold as measured with a tuning fork and an ABPI between 0.9 and 1.3), and a neuroischaemic group (presenting with ABPI < 0.9 and neuropathy).

Participants who presented with active ulceration or other significant comorbidities that could affect the distribution of thermographic patterns, such as rheumatoid arthritis or Raynaud's phenomenon, were excluded.

Following a 15-minute acclimatization period, all participants rested in a supine position on a couch in a room which was temperature controlled at 23°C.

An ABPI was obtained [14, 15], according to standard clinical practice utilizing a Huntleigh (Cardiff, Wales) Dopplex Assist. A cuff was applied proximal to the ankle, and an 8 Mhz doppler probe was applied at the posterior tibial artery and the dorsalis paedis artery. The probe was held at an angle of 45° against blood flow while the cuff was inflated until the doppler signal was cut off. Then the cuff pressure was slowly released. Once the signal was reobtained, the systolic pressure of the particular artery was noted.

This process was repeated for the brachial artery; the cuff was applied above the elbow and the doppler probe was held in order to obtain its systolic pressure. The ABPI was calculated with the higher of the posterior tibial and dorsalis paedis pressures being taken into consideration. Normal ABPI values ranged from 0.9 to 1.3.

Spectral Doppler waveform analysis was also employed to classify the recorded waveform as being triphasic, biphasic, or monophasic [16]. A triphasic waveform is indicative of normal arterial perfusion, whilst the other two classifications are indicative of PAD, with the monophasic waveform denoting a more severe form of the condition. Only those participants with monophasic waveforms and an ABPI < 0.6 were included to ensure an unequivocal diagnosis of PAD.

Testing for neuropathy involved the use of a 10 g Semmes Weinstein monofilament administered at 10 sites on each foot. In this validated method, exactly 10 g of force was applied before the monofilament bent, thus ensuring that exactly the same amount of force is applied. Neuropathy was diagnosed if at least one site was not felt by the participant. All the above measurements were carried out by the same experienced clinician in order to ensure consistency.

## 3. Image Acquisition, Segmentation, Data Extraction, and Analysis

A FLIR SC7200 infrared camera with a spatial resolution of 320 × 256 pixels and a temperature resolution of 20 mK was used for the acquisition of thermal images. The protocol for obtaining thermal images followed the recommendations of the American Academy of Thermology [17]. The camera was placed on a tripod 1.5 m from the subject and perpendicular to the body part that was being photographed [11]. Images of the plantar aspect of the feet were recorded for later analysis.

**Figure 1:**
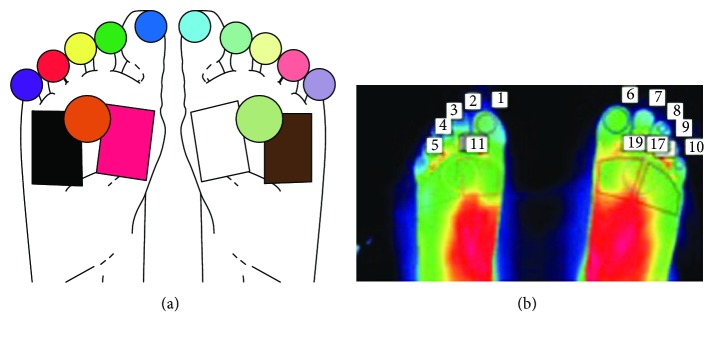
(a) Diagram showing the foot regions considered for temperature extraction. (b) An actual thermal image and the corresponding regions of interest. The temperatures from the toe regions and forefoot regions were considered for further analysis.

Thermal images obtained of the feet were divided into regions so that temperature data could be extracted (Figures 1(a) and 1(b)) [11].

## 4. Results

Thermographic images from 43 neuroischaemic limbs (from 30 subjects), 41 neuropathic limbs (from 32 subjects), 58 PAD limbs (from 42 subjects), 21 DM healthy limbs (15 subjects,), and 126 healthy limbs (from 63 subjects) were analyzed.

**Figure 2:**
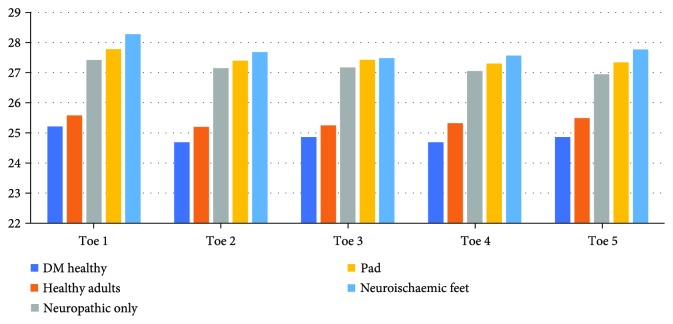
Toe temperature distribution.

**Figure 3:**
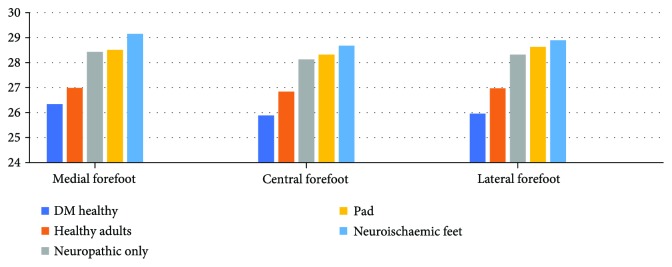
Forefoot temperature distribution.

When analyzing the mean temperature data in all 5 toes and 3 plantar regions of the forefoot (medial, central, and lateral regions) (Figures 2 and 3), there are significant differences in mean temperatures between the five groups of patients, as demonstrated by the Tukey test (Table 1). This test clusters these five groups into two groups where the mean temperatures of diabetic participants with peripheral arterial disease, diabetes patients with neuropathy, and diabetes patients with neuroischaemia are significantly higher than the mean temperatures of healthy adults and diabetes patients with no known complication. Thus, for further statistical comparison, these five categories were divided into a “healthy group” (comprised of healthy adults and DM participants with no known complications) and a “complications group” (comprised of neuropathic, neuroischaemic, and PAD participants).

## 5. Logistic Regression Analysis

In the first logistic regression model fit, we relate the health status (neuropathic, neuroischaemic, or PAD; healthy or DM healthy) to two predictors, which include toe temperature and toe location. As indicated in Table 2, a binomial distribution is assumed since the dependent variable has two categories, while a logit link function is used since this is the canonical link for the binomial distribution.

Toe location was not found to be a significant predictor since the *p* value (0.901) exceeds the 0.05 level of significance. A backward procedure was used to fit the parsimonious model, which identified temperature as a sole significant predictor.

**Figure 4:**
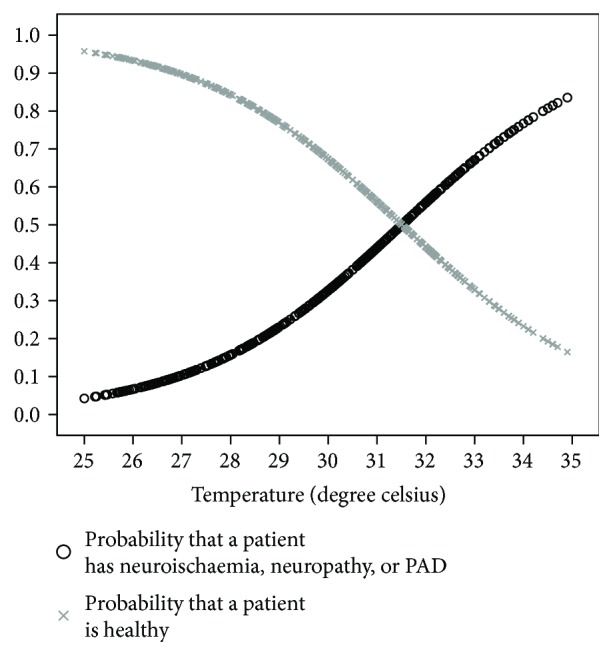
Logistical regression curves of toe temperatures.

As shown in Table 3, the regression coefficient of temperature (0.220) is positive indicating that the toe temperature of neuropathic, neuroischaemic, or PAD participants is expected to be higher than that of healthy or DM healthy patients. The odds ratio indicates that the odds that the participant has neuropathy, neuroischaemia, or PAD rather than being healthy increases by 24.7% for every 1°C increase in toe temperature. This odds ratio ranges from 19.8% to 29.7% assuming a 95% confidence level. The logistic regression model that yields the probability that a patient has neuropathy, neuroischaemia, or PAD given the toe temperature is given by

(1) $$\begin{array}{c} \log_{e}\left(\frac{p}{1-p}\right)=-5.786+0.220\;temperature, \end{array}$$

where *p* is the probability that the participant has neuropathy, neuroischaemia, or PAD and 1 − *p* is the probability that the patient is healthy. The probability curves displayed in Figure 4 clearly show that the likelihood of neuropathy, neuroischaemia, or PAD increases as the toe temperature increases.

In the second logistic regression model fit, we relate the health status (neuropathic, neuroischaemic, or PAD; healthy or DM healthy) to two predictors, which include temperature and forefoot location, whether medial, central, or lateral. A binomial distribution and a logit link function are again assumed.

Plantar location was not found to be a significant predictor since the *p* value (0.912) exceeds the 0.05 level of significance. A backward procedure was used to fit the parsimonious model, which identified temperature as a sole significant predictor (Table 4).

**Figure 5:**
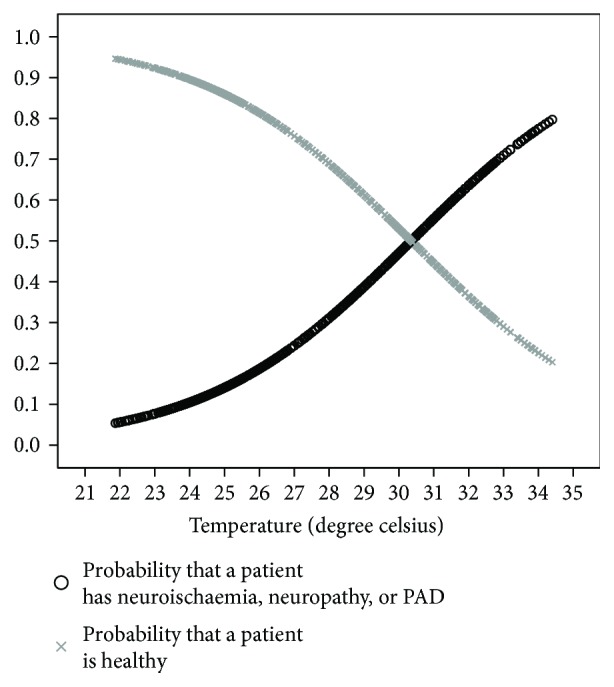
Logistical regression curves of forefoot temperatures.

The results in Table 5 indicate that the regression coefficient for the plantar forefoot temperature (0.254) is positive indicating that the plantar forefoot temperature of neuropathic, neuroischaemic, or PAD patients is expected to be higher than that of healthy or DM healthy patients. The odds ratio indicates that the odds that the participant has neuropathy, neuroischaemia, or PAD rather than being healthy increases by 28.9% for every 1°C increase in plantar temperature. This odds ratio ranges from 19.8% to 29.7% assuming a 95% confidence level. The logistic regression model that yields the probability that a patient has neuropathy, neuroischaemia, or PAD given the plantar temperature is given by

(2) $$\begin{array}{c} \log_{e}\left(\frac{p}{1-p}\right)=-6.949+0.254\;temperature, \end{array}$$

where *p* is the probability that the patient has neuropathy, neuroischaemia, or PAD and 1 − *p* is the probability that the patient is healthy. The probability curves displayed in Figure 5 clearly show that the likelihood of neuropathy, neuroischaemia, or PAD increases as the plantar forefoot temperature increases.

## 6. Discussion

The results of this thermographic study demonstrate three main inferences: (i) that there are no significant differences in mean temperatures of the toes and forefoot between healthy subjects and patients with diabetes showing no complications; (ii) that there are no significant differences in mean temperatures of these same areas between participants with complications of neuropathy, neuroischaemia, and PAD; (iii) that there are significantly higher mean temperatures in these latter group of subjects when compared to both healthy and DM participants with no complications.

This increase in temperature is further confirmed by the logistic regression models of both toe and forefoot areas, which establish temperature as being the sole significant predictor of complications. These models demonstrate that the probability of complications of PAD, neuropathy, and/or neuroischaemia being present increases as the temperature of these regions rises.

This study is the first of its kind to report temperature differences between possible categories of complications of DM relative to healthy adults. The authors recommend that these findings and thermographic techniques should be considered for further clinical investigations of the DM patient. These results imply that should a rise in temperature be detected in the diabetic foot, there is a higher likelihood that diabetic foot complications have set in, as further reported by Sun et al. [8] who state that thermographic patterns may change as early as one week prior to ulceration. The findings of the study indicate that an increase in temperature may not necessarily imply impending ulceration, but simply the development of peripheral neuropathy, ischaemia, or both.

Further research is warranted to establish whether the inclusion of thermography into screening protocols could help detect the development of diabetic foot complications earlier so that appropriate prompt preventative measures may be taken to avoid unnecessary complications.

Whilst neuropathic feet have been previously reported as being warmer than healthy feet, we can now confirm that even neuroischaemic and ischaemic feet exhibit the same trend. This may be due to altered thermoregulatory mechanisms of the feet, which can be affected by both neuropathy and PAD. Local ischaemia may lead to disruption of sympathetically mediated noradrenergic vasoconstriction which leads to increased flow to the cutaneous vessels rather than through the deeper nutritive vessels which in turn leads to higher heat emissivity. Arteriovenous anastamoses (AVA) which are thick-walled, low resistance conduits allow high-flow rates directly from arterioles to venules. AVA are numerous and richly innervated by sympathetic vasoconstrictor nerves. Substantial changes occur in blood flow depending on whether AVAs are closed or open [18].

It is reported that the application of clinical examination or nerve conduction studies alone is not adequate in screening diabetic at-risk feet at early stage [8]; thus, the use of an adjunct method such as thermography may prove useful.

Further research into thermographic patterns of patients with diabetes and with active ulcers may help elucidate the natural history of development of ulceration.

## 7. Conclusions

This study has confirmed that the mean temperatures of the toes and forefeet of the complications group exhibit significantly higher temperatures than those of the healthy group, whilst each group presents with comparable temperatures within themselves. These results indicate that thermography demonstrates potential as a screening or clinical investigation tool, although more research in the area is warranted.

## Figures and Tables

**Table 1:** Tukey test to compare mean temperatures between the two groups.

		Sample size	Mean	Std. deviation	*p* value
Toe 1	Neuropathic, neuroischaemic, and PAD	121	27.83	2.637	0.000
	Healthy and DM healthy	123	25.52	3.473	
Toe 2	Neuropathic, neuroischaemic, and PAD	128	27.38	2.899	0.000
	Healthy and DM healthy	122	25.12	3.452	
Toe 3	Neuropathic, neuroischaemic, and PAD	131	27.33	2.886	0.000
	Healthy and DM healthy	123	25.19	3.476	

Toe 4	Neuropathic, neuroischaemic, and PAD	125	27.27	2.926	0.000
	Healthy and DM healthy	123	25.21	3.317	
Toe 5	Neuropathic, neuroischaemic, and PAD	125	27.29	2.852	0.000
	Healthy and DM healthy	123	25.38	3.238	
Medial forefoot	Neuropathic, neuroischaemic, and PAD	135	28.67	2.454	0.000
	Healthy and DM healthy	123	26.88	2.874	

Central forefoot	Neuropathic, neuroischaemic, and PAD	134	28.35	2.474	0.000
	Healthy and DM healthy	123	26.68	2.835	
Lateral forefoot	Neuropathic, neuroischaemic, and PAD	130	28.58	2.374	0.000
	Healthy and DM healthy	123	26.80	2.861	

**Table 2:** Likelihood ratio tests (model 1).

Likelihood ratio tests				
Effect	Model fitting criteria	Likelihood ratio tests		
	−2 log likelihood	Chi-square	df	*p* value
Intercept	1495.659	0.000	0	.
Temperature	1634.676	139.02	1	0.000
Toe location	1496.714	1.055	4	0.901

**Table 3:** Parameter estimates of the logistic regression model for toe temperatures.

Effect	B	Std. error	Wald	*p* value	Odds ratio	95% CI for odds ratio	
						Lower bound	Upper bound
Intercept	−5.786	0.534	117.363	0.000			
Temperature	0.220	0.020	119.693	0.000	1.247	1.198	1.297

**Table 4:** Likelihood ratio tests (model 2).

Likelihood ratio tests				
Effect	Model fitting criteria	Likelihood ratio tests		
	−2 log likelihood	Chi-square	df	*p* value
Intercept	924.056	0.000	0	.
Temperature	1004.520	80.464	1	0.000
Plantar forefoot location	924.240	0.184	2	0.912

**Table 5:** Parameter estimates of the logistic regression model for forefoot temperatures.

Effect	B	Std. error	Wald	*p* value	Odds ratio	95% CI for odds ratio	
						Lower bound	Upper bound
Intercept	−6.949	0.846	67.395	0.000			
Temperature	0.254	0.030	69.481	0.000	1.289	1.214	1.368
